# Highly sensitive two-dimensional MoS_2_ gas sensor decorated with Pt nanoparticles

**DOI:** 10.1098/rsos.181462

**Published:** 2018-12-12

**Authors:** Jaeseo Park, Jihun Mun, Jae-Soo Shin, Sang-Woo Kang

**Affiliations:** 1 Advanced Instrumentation Institute, Korea Research Institute of Standards and Science, Daejeon 34113, Republic of Korea; 2 Science of Measurement, University of Science and Technology, Daejeon 34113, Republic of Korea; 3 Department of Advanced Materials Engineering, Daejeon University, Daejeon 34520, Republic of Korea

**Keywords:** molybdenum disulfide, transition metal dichalcogenides, gas sensor, Pt nanoparticles

## Abstract

A two-dimensional molybdenum disulfide (MoS_2_)-based gas sensor was decorated with Pt nanoparticles (NPs) for high sensitivity and low limit of detection (LOD) for specific gases (NH_3_ and H_2_S). The two-dimensional MoS_2_ film was grown at 400°C using metal organic gas vapour deposition. To fabricate the MoS_2_ gas sensor, an interdigitated Au/Ti electrode was deposited using the electron beam (e-beam) evaporation method with a stencil mask. The MoS_2_ gas sensor without metal decoration sensitively detects NH_3_ and H_2_S gas down to 2.5 and 30 ppm, respectively, at room temperature (RT). However, for improved detection of NH_3_ and H_2_S gas, we investigated the functionalization strategy using metal decoration. Pt NP decoration modulated the electronic properties of MoS_2_, significantly improving the sensitivity of NH_3_ and H_2_S gas by 5.58× and 4.25×, respectively, compared with the undecorated MoS_2_ gas sensor under concentrations of 70 ppm. Furthermore, the Pt NP-decorated MoS_2_ sensor had lower LODs for NH_3_ and H_2_S gas of 130 ppb and 5 ppm, respectively, at RT.

## 1. Introduction

Metal oxide-based gas sensors possess many merits, such as relatively high sensitivity and low cost, which has garnered much attention to the material [1]. However, to obtain such gas-sensing properties for a specific gas, these gas sensors generally suffer from several issues, such as thermal instability and high power consumption, due to poor performance at low operating temperatures [1]. As gas detection relies strongly on the properties of material, new materials that possess excellent characteristics for adsorption of gas molecules at low operating temperatures are in great demand as a way to overcome these deficiencies.

Among the new sensing materials, the two-dimensional materials [2–22] with novel and unique electronic, optical and mechanical properties have attracted much attention. In addition to their excellent properties, the novel two-dimensional gas-sensing materials possess structural advantages, such as a high surface-to-volume ratio and the tunable functionality of the surface for decoration species or functional groups, so they have attracted immense interest as new gas-sensing materials [2–4] that can sensitively react upon exposure to a lower level of a specific gas at low temperature. Even though graphene [5–7]—with its outstanding characteristics—is the most widely known two-dimensional material, transition metal dichalcogenides (TMDCs) [8–13,15–22] have great potential to serve as new materials for advanced electronic devices owing to their excellent characteristics, such as high electron mobility, high flexibility, high elasticity and low power consumption. The charge transfer mechanism between gas molecules and molybdenum disulfide (MoS_2_)—which is a typically known two-dimensional TMDC—was previously reported for gas detection [15]. In addition, to improve the performance of the gas sensor, various studies on functionalization using metal [23–30] or metal oxide [31,32] and structural modification [17,33] have been reported. Especially, functionalization [23–32] using metal decoration [23–30] on two-dimensional materials can open an avenue for gas detection [23–32].

In this work, we investigated the performance of a two-dimensional MoS_2_-based gas sensor and the effects of functionalization using Pt nanoparticle (NP) decoration. Metal organic chemical vapour deposition (MOCVD), which can control the number of two-dimensional material layers and synthesize scalable two-dimensional-layered materials with high quality, was employed to grow the two-dimensional MoS_2_ film. Moreover, MOCVD is capable of synthesizing high-quality two-dimensional MoS_2_ directly on the sensor substrate to easily fabricate the MoS_2_ gas sensor. The MoS_2_ sensor consisted of the MOCVD-grown MoS_2_ film as the active channel and an interdigitated Au/Ti electrode fabricated using the electron beam (e-beam) evaporation method with a stencil mask. At room temperature (RT), the MoS_2_ sensor could sensitively detect down to 2.5 and 30 ppm for NH_3_ and H_2_S gas, respectively. However, the detection of NH_3_ and H_2_S gas still requires higher sensitivity and a lower limit of detection (LOD) owing to the inhalation toxicology of both gases.

Because both gases exhibit inhalation toxicology that leads to lung and organ injury or even death as well as skin irritation, several international standards have been developed as guidelines to assist in the control of these health hazards. For example, the permissible exposure limits, i.e. regulatory limits, in the work place stipulated by the Occupational Safety and Health Administration (OSHA) are 50 ppm for NH_3_ and 10 ppm for H_2_S. However, in many cases, the recommended limits of NH_3_ and H_2_S gas, which are characterized by an extremely irritating odour, lie below the regulatory limit stipulated by OSHA, so studies for high sensitivity and low LOD are still required. Thus, we investigated a functionalization strategy, i.e. metal decoration, to obtain excellent gas-sensing characteristics (e.g. higher sensitivity and lower LOD) for NH_3_ and H_2_S gas. To improve the sensitivity and LOD for NH_3_ and H_2_S gas, which act as electron donors on the MoS_2_, Pt metal NPs, which resist corrosion and oxidation, yielding stable doping as a noble metal [27], are used as the decorating material. Furthermore, Pt NPs double the p-type doping effect compared with Au NPs [27], which commonly act as a p-dopant [12], so Pt NPs could improve the sensing characteristics for NH_3_ and H_2_S gas. Even though various studies have already been reported [15–19,34] on the detection of various toxic gases (e.g. NO_2_, NH_3_, H_2_ and CO_2_) using two-dimensional TMDC-based sensors, most use flake or bulk two-dimensional TMDC instead of two-dimensional MoS_2_ film at RT. In this work, H_2_S detection using a two-dimensional MoS_2_ sensor in addition to the detection of well-known gases (e.g. NH_3_, NO_2_, H_2_ and volatile organic compounds) is reported. The H_2_S response is lower than the NH_3_ response on the MoS_2_ sensor, confirming that the MoS_2_ sensor has lower charge transfer for the detection of H_2_S than NH_3_ gas. Compared to an undecorated MoS_2_ sensor, the Pt NP-decorated MoS_2_ sensor detected NH_3_ and H_2_S gas with high sensitivity down to 600 ppb and 5 ppm, respectively. In addition, NH_3_ detection experiments were conducted at concentrations of 600 ppb or less in order to determine the LOD of the Pt-decorated MoS_2_ sensor for NH_3_ gas.

## 2. Material and methods

### 2.1. MOCVD growth of two-dimensional MoS_2_ monolayer film with bilayer islands

As previously reported [35], the two-dimensional MoS_2_ film was grown using MOCVD. The MoS_2_ film was grown by a showerhead-type reactor using Mo(CO)_6_ (better than 99.9% purity, Sigma-Aldrich), high-purity H_2_ (99.999%), Ar (99.999%) and H_2_S (99.9%) with an operating temperature of 400°C. A p-type silicon wafer (1–10 Ω cm) with 300 nm silicon dioxide was used as the substrate for growth. The piranha solution (H_2_SO_4_ : H_2_O_2_ = 3 : 1) was used for pretreatment to passivate dangling bonds [35] and make a hydrophilic substrate, resulting in the control of more nucleation sites on the substrate; the piranha treatment was performed by immersing the substrate in the piranha solution for 10 min. After immersion in the piranha solution, the substrate was thoroughly rinsed in deionized water through immersion and sonication, and it was blow-dried with high-purity N_2_ (99.999%). After cleaning this, the substrate was placed on a silicon carbide-coated graphite susceptor and immediately loaded into a load-lock chamber, which was connected with the MOCVD process chamber to prevent any contamination. Subsequently, the substrate on the graphite susceptor was loaded into the process chamber to grow the two-dimensional MoS_2_ film. MoS_2_ film was grown using sublimed Mo(CO)_6_ precursor vapour with high-purity H_2_S, Ar and H_2_ flow for high-quality MoS_2_ at 300°C by the reaction between sulfur and gas with the Mo-precursor under a pressure of 5 Torr. Consequently, the fully covered monolayer MoS_2_ film was synthesized with bilayer islands without empty spaces of MoS_2_.

### 2.2. Fabrication of MoS_2_-based devices and decoration with metal NPs

**Figure 1.**
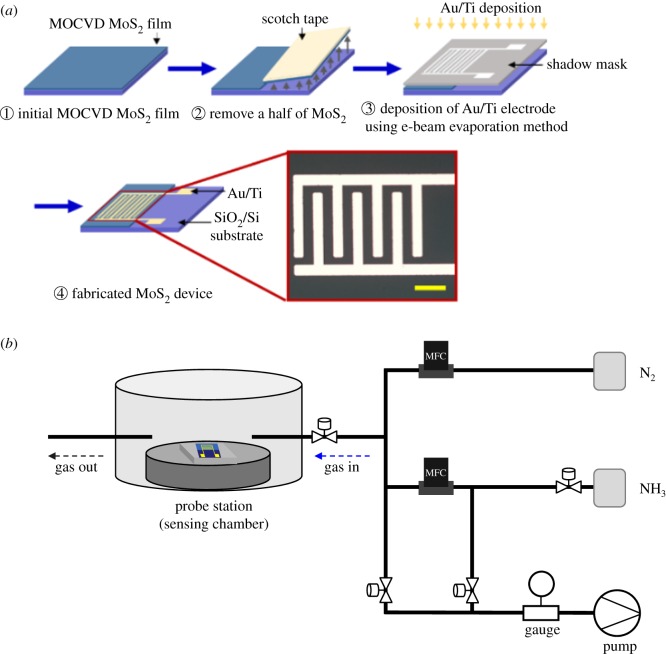
(*a*) Schematic illustration of the fabrication of MoS_2_ gas sensor and optical image of interdigitated electrode on the MoS_2_. The scale bar is 50 µm. (*b*) Schematic illustration of the gas-sensing system.

To prevent any contamination by chemical substances during the fabrication of the MoS_2_ gas sensor, we chose a solution-free process, as shown in figure 1*a*. The MoS_2_ gas sensor, which consisted of the MOCVD two-dimensional MoS_2_ active channel and the interdigitated electrode, was fabricated using the e-beam evaporation method with a stencil mask. To use the MoS_2_ as the active material of the gas sensor, half of the MoS_2_ film was removed by the scotch-tape method. The interdigitated electrode was patterned on the remaining MoS_2_ film using the e-beam evaporation method and a stencil mask with the interdigitated pattern. Additionally, the electrode pad for resistance measurement was patterned on Si with SiO_2_ where the MoS_2_ was removed. The interdigitated electrode region was made from 5 nm Ti and 50 nm Au using an e-beam evaporation method under a pressure of approximately 10^−7^ Torr. Figure 1*a* also shows an optical image of the active channel (MoS_2_ film, 70 µm in width) and the interdigitated Au/Ti electrode of the MoS_2_ gas sensor. The active channel material detects gas molecules, and the interdigitated electrode collects charge. In this work, two MoS_2_-based devices (one decorated with Pt NPs and one without Pt NPs) were made to investigate the effect of Pt NP decoration. The e-beam evaporation method was used to randomly distribute Pt NPs on the MoS_2_. The Pt NPs (approx. 1 nm in thickness) were monitored with a crystal thickness sensor of e-beam evaporation under a pressure of approximately 10^−7^ Torr.

### 2.3. Gas-sensing experiments

A gas-sensing system that allows the users to customize experimental conditions according to their need was set up, as shown in figure 1*b*. The system consisted of analyte gas (NH_3_ and H_2_S), dilution (or purging) gas (N_2_), a pneumatic valve for opening/closing gas flow, a mass flow controller for regulating analyte gas and dilution gas, a sensing chamber for the gas detection process and a rotary pump for purging the atmosphere after gas-sensing measurements. The gas-sensing experiments were conducted under analyte gas (NH_3_ or H_2_S) diluted with N_2_ in a sensing chamber, and the concentration of analyte gas was controlled by modulating the flow rate of analyte gas and N_2_ dilution gas. The fabricated MoS_2_ gas sensor was placed in the sensing chamber for gas detection. After loading one of the MoS_2_ gas sensors (i.e. bare MoS_2_ or Pt-decorated MoS_2_), the gas-sensing system was sufficiently stabilized under N_2_ gas in the chamber and then the gas-sensing experiments were repeatedly performed by automatically controlling the purging step and analysis step for 10 and 5 min, respectively. The experiments were done with analyte concentrations of 600 ppb and 1, 2.5, 5, 15, 30 and 70 ppm. To measure the LOD of NH_3_ gas for the Pt-decorated MoS_2_ sensor, the experiments were done with concentrations of 130 ppb. The resistance value of the MoS_2_ sensor was measured with a Keysight B2985A high-resistance meter. Because the MoS_2_ gas sensor has excellent gas-sensing characteristics at high operating temperatures and RT, all experiments were performed at RT under ambient pressure.

## 3. Results and discussion

**Figure 2.**
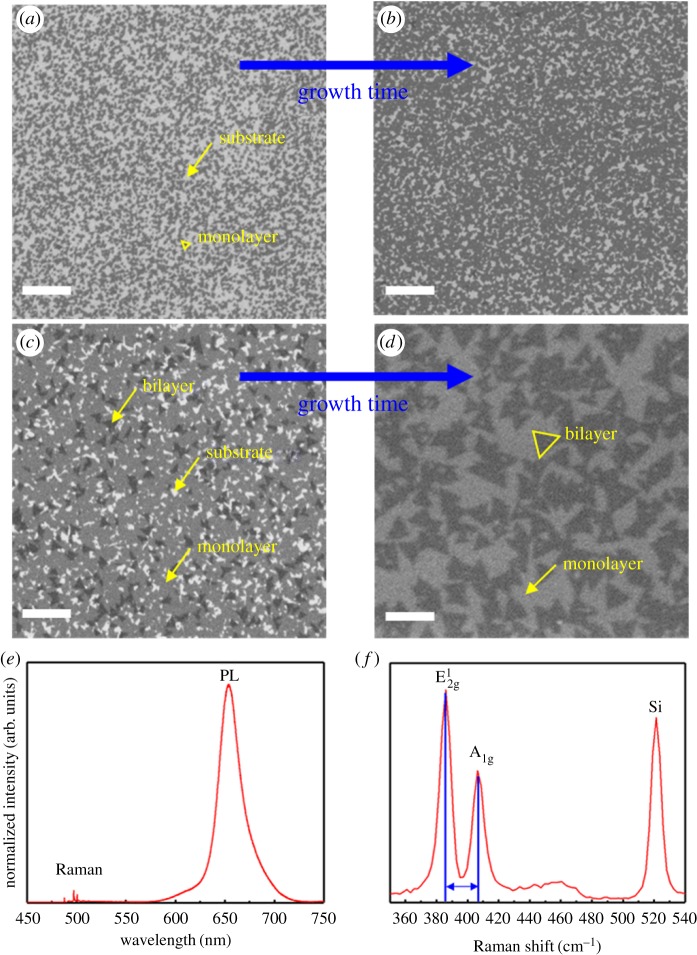
Characteristics of as-grown two-dimensional MoS_2_. (*a*–*d*) SEM images. The scale bar is 300 nm. (*e*) Photoluminescence. (*f*) Raman spectrum.

To examine the morphology and grain size of the as-grown MoS_2_, the MoS_2_ film was characterized using scanning electron microscopy (SEM) (Hitachi S-4800), as shown in figure 2*a*–*d*: figure 2*a* shows smaller monolayer MoS_2_ clusters (growth time: 1 h), figure 2*b* shows larger monolayer MoS_2_ clusters with a little bit of space (growth time: 2 h) and figure 2*c* shows monolayer MoS_2_ with bilayer islands (growth time: 3 h), which were obtained by controlling coverage using the growth time without a change of other process conditions. As the growth time increased, the coalescence of these monolayer islands led to a fully covered MoS_2_ film. Under a highly strong sulfiding condition, the layer atoms are more strongly attracted to the substrate than to themselves, thereby accelerating two-dimensional growth. [35] Many small nucleation sites of MoS_2_ were made on the SiO_2_/Si substrate by pretreatment with the piranha solution, as shown in figure 2*a,b*. Triangular monolayer MoS_2_ clusters with S-edge termination are more stable, and figure 2*a,b* shows the initially grown triangular MoS_2_ monolayer islands on the SiO_2_/Si substrate. Before the monolayer MoS_2_ film had fully formed, bilayer islands began to grow on the merging monolayer islands of MoS_2_ on the substrate. After sufficient growth of the MoS_2_, the fully formed monolayer MoS_2_ film with bilayer islands was deposited after a growth time of 4 h 30 min, as shown in figure 2*d*. Figure 2*d* shows that a monolayer two-dimensional MoS_2_ film with bilayer islands was grown using the MOCVD method. Thus, MOCVD two-dimensional MoS_2_ was successfully grown at 300°C by the reaction between the sulfur and gas reaction with the Mo-precursor under the sulfiding conditions. The grain size of the bilayer islands on the fully formed monolayer film was about 100 nm. Because the adsorption of gas molecules can be attributed to the influence of the many activation sites caused by the presence of the many edges site of the bilayer islands, the fully formed monolayer MoS_2_ film with bilayer islands was used as the active material of the gas sensor in this work.

To examine the number of the MoS_2_ layers, photoluminescence and Raman spectroscopy (Renishaw inVia Raman microscope) were used. A laser with an excitation wavelength of 488 nm was used. The spectra were also normalized at the Si peak (520.7 cm^–1^). As a result, the photoluminescence spectrum of the MoS_2_ has an adsorption peak at 659 nm (1.88 eV). The Raman spectrum represents the in-plane vibration (E^1^_2 g_) and the out-of-plane vibration (A_1 g_) from the MoS_2_. The peak position difference between E^1^_2 g_ and A_1 g_ was 21.81 cm^–1^, which indicates that the MoS_2_ has two layers based on the laser wavelength.

**Figure 3.**
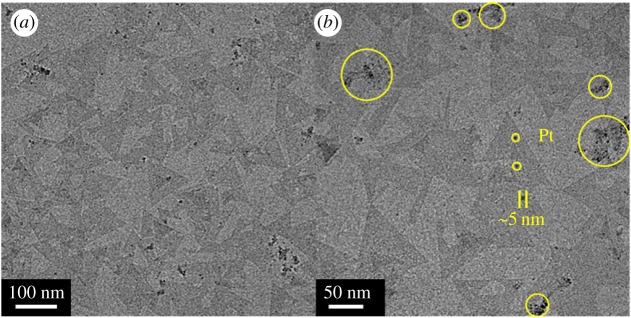
TEM images of the Pt : MoS_2._

To investigate the effects of Pt NP decoration, we made two sensors: an undecorated MoS_2_ sensor and a Pt NP-decorated MoS_2_ sensor. Henceforth, the undecorated MoS_2_ and Pt NP-decorated MoS_2_ sensors will be denoted as bare MoS_2_ and Pt : MoS_2_, respectively. Before the gas-sensing experiments, a transmission electron microscope (TEM) (FEI Tecnai G2 F30 S-Twin) was used to characterize the morphology and structure of the as-obtained Pt : MoS_2_. The decorated Pt NPs on MoS_2_ were confirmed by TEM images. As shown in figure 3, a number of Pt NPs indicated by yellow circles and the TEM image clearly show small NPs with a diameter around 5 nm. The TEM image of the Pt : NP-decorated MoS_2_ reveals the monolayer MoS_2_ film (brightest), the triangular bilayer islands (brighter) and the Pt NPs (darker). Moreover, since Pt NPs were physically decorated by the e-beam evaporation method, the TEM image showed that the Pt NPs were randomly distributed on the MoS_2_.

**Figure 4.**
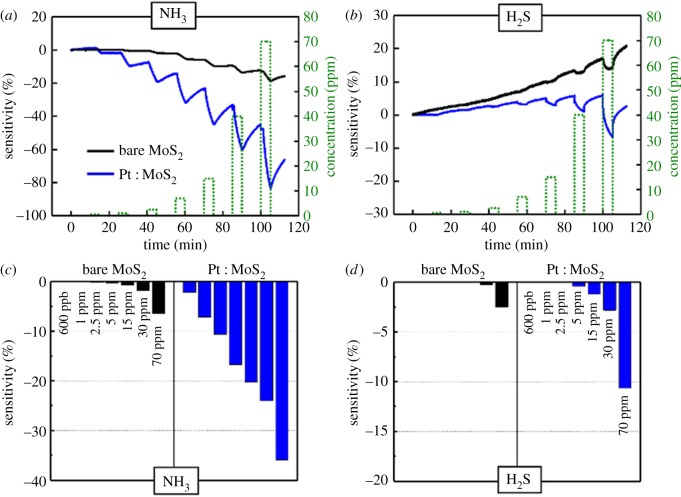
Gas-sensing characteristics of the MoS_2_ gas sensor (*a*,*c*) Transient NH_3_ or H_2_S gas-sensing characteristics. (*b*,*d*) Gas sensitivity summary of NH_3_ or H_2_S of the bare MoS_2_ and Pt : MoS_2_ gas sensor.

**Figure 5.**
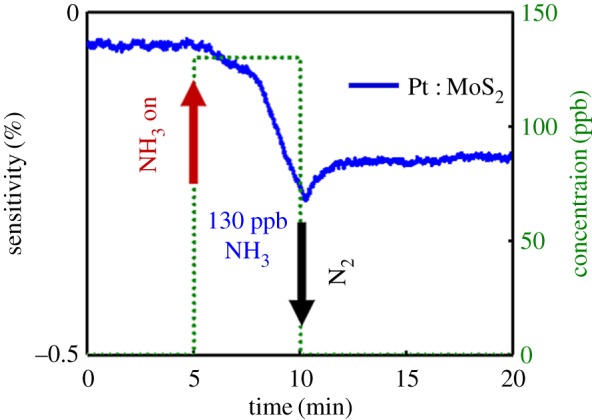
The Pt : NP-decorated MoS_2_ gas sensor response upon exposure to 130 ppb.

**Figure 6.**
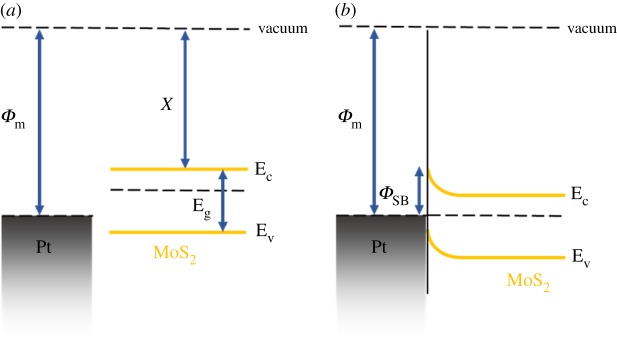
Pt : MoS_2_ energy diagram. (*a*) Before contact. (*b*) After contact. The *χ* is the electron affinity of MoS_2_. The *Φ*_m_ is the work function of Pt metal.

The transient NH_3_ or H_2_S gas-sensing characteristics of the two MoS_2_ gas sensors are shown in figure 4. With incrementally increasing concentrations (600 ppb and 1, 2.5, 5, 15, 30 and 70 ppm) of analyte gas, the gas responses of two sensors were investigated under NH_3_ and H_2_S gas. The sensitivity to the analyte gas was calculated using Δ*R*/*R*_p_ = (*R*_a_ − *R*_p_)/*R*_p_, where *R*_p_ and *R*_a_ represent the resistances of the device under the N_2_ gas (purging gas) and the analyte gas, respectively. When exposed to the NH_3_ or H_2_S gas, the MoS_2_ sensors showed decreasing resistance (negative sensitivity). This means that both NH_3_ and H_2_S gas act as electron donors, transferring electrons to the conduction band of MoS_2_ [15]. Thus, this leads to increased electron concentration and conductivity, and the resulting n-doping brings the Fermi level closer to the conduction band edge. Figure 4*a* shows that the LOD of NH_3_ gas was as low as 2.5 ppm and the absolute sensitivity of NH_3_ gas ranged from 2.5 to 70 ppm on the bare MoS_2_ gas sensor at RT. Moreover, the LOD of H_2_S gas was as low as 30 ppm, and the sensitivity of H_2_S gas ranged from 30 to 70 ppm at RT, as shown in figure 4*b*. The two-dimensional MoS_2_ gas sensor is the first film sensor to exhibit H_2_S gas-sensing characteristics (figure 4*b*). We concluded that detection of H_2_S gas can be attributed to the influence of many activation sites caused by the presence of bilayer islands on the MoS_2_ gas sensor. As the concentration of analyte gas (NH_3_ or H_2_S) increased, the absolute sensitivity of the MoS_2_ sensor increased at RT (figure 4*c,d*). Because the bare MoS_2_ gas sensor has higher sensitivity and lower LOD for NH_3_ than H_2_S gas sensor, it experiences a smaller charge transfer from H_2_S than from NH_3_. To confirm the LOD of NH_3_ gas for the Pt : MoS_2_ gas sensor, we performed an additional gas-sensing experiment under analyte concentrations of 600 ppb or less. This experiment demonstrates that the Pt : MoS_2_ gas sensor has an LOD of NH_3_ gas down to 130 ppb, as shown in figure 5. Table 1 summarizes the gas-sensing characteristics of many TMDC-based gas sensors for NH_3_ or H_2_S gas at RT. It shows that the Pt : MoS_2_ gas sensor has high sensitivity and low LOD for both NH_3_ and H_2_S gas. Figure 6 shows a schematic of the energy band diagram of MoS_2_ and Pt, where the work function (*Φ*_m_) of Pt is approximately 5.65 eV [36] and the electron affinity (*χ*) of MoS_2_ is approximately 4.2 eV [37]. Metal decoration is generally known to enhance sensitivity to a target gas, and Pt NPs act as a p-dopant, increasing sensitivity to gases that act as electron donors on the MoS_2_ sensor. Overall, the Pt : MoS_2_ sensor reduced the LOD of NH_3_ and H_2_S gas to 130 ppb and 5 ppm, respectively, which are far lower than those of the bare MoS_2_ sensor (2.5 and 30 ppm, respectively). Specifically, the Pt : MoS_2_ gas sensor exhibited 5.58× improved NH_3_ sensitivity under a concentration of 70 ppm, and the H_2_S sensitivity improved by 4.25× at a concentration of 70 ppm. Even though all gas-sensing experiments were performed at RT, the bare MoS_2_ and Pt : MoS_2_ gas sensors exhibited excellent sensing characteristics for the target gases (NH_3_ and H_2_S).

**Table 1.** A summary of two-dimensional TMDC-based gas sensor for NH_3_ or H_2_S detection at RT.

no.	method of synthesis	sensing material	target gas	LOD (ppm)	operating temperature (°C)	ref.
1	CVD	MoS_2_ three layer film	NH_3_	5	RT	[15]
2	CVD	MoS_2_ one layer film	NH_3_	1	RT	[16]
3	magnetron sputtering	MoS_2_nanostructure	NH_3_	10	RT	[17]
4	exfoliated	MoSe_2_ one layer	NH_3_	50	RT	[18]
5	exfoliated	MoTe_2_ few layer	NH_3_	2	RT	[19]
6	exfoliated	WS_2_ nanowire–nanoflake hybrid	NH_3_	5	30	[20]
7	ALD	WSe_2_ three layer film	NH_3_	20	RT	[21]
8	MOCVD	Pt-decorated MoS_2_ one layer film with bilayer islands	NH_3_	0.13	RT	This work
9	exfoliated	WS_2_ nanowire–nanoflake hybrid	H_2_S	1	30	[20]
10	exfoliated	WSe_2_ 50 layer flake	H_2_S	1	RT	[22]
11	MOCVD	Pt-decorated MoS_2_ one layer film with bilayer islands	H_2_S	5	RT	This work

## 4. Conclusion

In summary, Pt NPs were decorated on MOCVD two-dimensional MoS_2_ to obtain high sensitivity and low LOD for NH_3_ and H_2_S gases. The electronic properties of MoS_2_ were tuned through decoration with Pt NPs, improving the sensitivity and lowering the LOD of the gas sensor for the target gases. The Pt NPs act as a p-dopant, depleting the electron carriers of the MoS_2_ film, thereby improving the sensitivity to NH_3_ and H_2_S gas by 5.58× and 4.25×, respectively, compared to the bare MoS_2_ gas sensors under concentrations of 70 ppm. The Pt : MoS_2_ gas sensor exhibited LODs of 130 ppb and 5 ppm for NH_3_ and H_2_S gas, respectively, which are far lower than those for the bare MoS_2_ gas sensor (2.5 and 30 ppm, respectively). Even though all gas-sensing experiments were carried out at RT, the MoS_2_-based gas sensors showed excellent gas-sensing characteristics. Therefore, functionalization using metal decoration on a two-dimensional gas sensor can improve the sensitivity and LOD for a specific gas by modulating the electronic properties of the two-dimensional material. This strategy opens an avenue for effective toxic gas detection with two-dimensional gas sensors.

## Data accessibility

This article does not contain any additional data and all data are provided in the main text.
